# Phenotype of genetically confirmed Silver-Russell syndrome beyond childhood

**DOI:** 10.1136/jmedgenet-2019-106561

**Published:** 2020-02-13

**Authors:** Oluwakemi Lokulo-Sodipe, Lisa Ballard, Jenny Child, Hazel M Inskip, Christopher D Byrne, Miho Ishida, Gudrun E Moore, Emma L Wakeling, Angela Fenwick, Deborah J G Mackay, Justin Huw Davies, I Karen Temple

**Affiliations:** 1 Human Development and Health, Faculty of Medicine University of Southampton, Southampton, UK; 2 Department of Paediatric Endocrinology, University Hospital Southampton NHS Foundations Trust, Southampton, UK; 3 Cancer Sciences, University of Southampton Faculty of Medicine, Southampton, UK; 4 Child Growth Foundation, Sutton Coldfield, Birmingham, UK; 5 MRC Epidemiology Unit, University of Southampton Faculty of Medicine, Southampton, UK; 6 NIHR Southampton Biomedical Research Centre, University Hospital Southampton NHS Foundation Trust, Southampton, UK; 7 Great Ormond Street Institute of Child Health, University College London, London, UK; 8 Great Ormond Street Hospital for Children NHS Foundation Trust, London, UK; 9 Clinical Ethics and Law at Southampton (CELS), Faculty of Medicine University of Southampton, Southampton, UK; 10 Wessex Regional Genetics Laboratory, Salisbury Hospital NHS Foundation Trust, Salisbury, Wiltshire, UK; 11 Department of Paediatric Endocrinology, University Hospital Southampton NHS Foundation Trust, Southampton, UK; 12 Wessex Clinical Genetics Service, University Hospital Southampton NHS Foundation Trust, Southampton, UK

**Keywords:** imprinting, Silver Russell syndrome, short stature, uniparental disomy

## Abstract

**Background:**

Silver-Russell syndrome is an imprinting disorder that restricts growth, resulting in short adult stature that may be ameliorated by treatment. Approximately 50% of patients have loss of methylation of the imprinting control region (H19/IGF2:IG-DMR) on 11p15.5 and 5%–10% have maternal uniparental disomy of chromosome 7. Most published research focuses on the childhood phenotype. Our aim was to describe the phenotypic characteristics of older patients with SRS.

**Methods:**

A retrospective cohort of 33 individuals with a confirmed molecular diagnosis of SRS aged 13 years or above were carefully phenotyped.

**Results:**

The median age of the cohort was 29.6 years; 60.6% had a height SD score (SDS) ≤−2 SDS despite 70% having received growth hormone treatment. Relative macrocephaly, feeding difficulties and a facial appearance typical of children with SRS were no longer discriminatory diagnostic features. In those aged ≥18 years, impaired glucose tolerance in 25%, hypertension in 33% and hypercholesterolaemia in 52% were noted. While 9/33 accessed special education support, university degrees were completed in 40.0% (>21 years). There was no significant correlation between quality of life and height SDS. 9/25 were parents and none of the 17 offsprings had SRS.

**Conclusion:**

Historical treatment regimens for SRS were not sufficient for normal adult growth and further research to optimise treatment is justified. Clinical childhood diagnostic scoring systems are not applicable to patients presenting in adulthood and SRS diagnosis requires molecular confirmation. Metabolic ill-health warrants further investigation but SRS is compatible with a normal quality of life including normal fertility in many cases.

## Introduction

Silver-Russell syndrome (SRS)^1 2^ is an imprinting disorder associated with restricted growth. A recently published study has demonstrated a live birth SRS prevalence of 1/15 866,^3^ which is higher than previous estimates.^4^ Growth restriction in utero affects birth length and weight, with relative head sparing. Malnutrition, defined as weight/expected weight-for-height ratio <80%, is described in 70% of children with SRS and a body mass index (BMI) SD score (SDS) of <−2 in 61%.^5^ Absence of catch-up growth results in reduced adult height, although height can be improved by treatment with growth hormone (GH).^6^ Developmental delay is reported in some cases and varies from mild transient speech or motor delay to more severe developmental and behavioural phenotypes.^5 7^

In 65% of patients with SRS, an underlying molecular aberration can be detected, affecting imprinted and non-imprinted fetal growth factors and their cellular networks; 5%–10% of individuals have maternal uniparental disomy of chromosome 7 (matUPD7),^8^ and around 50% have loss of methylation (LOM) of the telomeric imprinting control region (H19/IGF2:IG‐DMR or imprinting centre 1 (IC1)) on 11p15.5.^9^ Rare cases have chromosome rearrangements involving IC1^10^ or mutations in genes in the IGF2 pathway (*HMGA2, PLAG1* and *IGF2*) or *CDKN1C*.^11–13^ The heterogeneity in molecular aetiology partially explains differences in clinical presentation: for example, body asymmetry (tissue hypotrophy) and congenital anomalies (eg, hypospadias, uterine malformations) are more commonly associated with IC1 LOM,^7^ whereas heritable genetic changes underlie some familial cases,^11 14^ and verbal dyspraxia and dystonia/tremor are recognised in some cases with matUPD7.^7^ There is clinical overlap with other imprinting disorders (eg, Temple syndrome due to imprinting errors on chromosome 14,^15^ matUPD20 syndrome^16^ and IMAGe syndrome^17^), therefore the ‘clinical diagnosis’ of SRS, reported prior to current genetic stratification, is likely to include considerable heterogeneity. Furthermore, many clinical features of SRS are non-specific, variable and age-dependent, challenging diagnosis and potentially underestimating prevalence.

Common childhood features of SRS crystallise into six key findings: birth weight and/or birth length ≤ −2 SDS, height at 2 years of ≤ −2 SDS, relative macrocephaly at birth, body asymmetry, protruding forehead and significant feeding difficulties in childhood. These features form the basis of the Netchine-Harbison clinical scoring system (NHCSS, see ‘Methods’ section),^18^ a useful diagnostic tool for children with unexplained short stature.^19^

The classical facial features of SRS (triangular facial shape, prominent forehead, relative macrocephaly, micrognathia, down-turned corners of the mouth) become less obvious with increasing age.^7 20^ In historical cohorts where adults are included, height rather than weight is predominantly described. For example, mean height SDS of −3.58 and −3.61 in boys and girls, respectively was reported in 18 children with clinically diagnosed SRS approaching final height^21^; mean adult heights in 368 clinically diagnosed men and women were 151.2 cm (−3.7 SDS) and 139.7 cm (−4.2 SDS), respectively.^22^ Such reports may have contributed to a general assumption that individuals with SRS have a minimal requirement for medical input once adult height is attained. Many individuals with SRS are lost to follow-up at the time of transition to adult services. Educational attainment and employment have been reported inconsistently.

Medical problems in older individuals with SRS have been reported mainly from single case reports; findings include dilated cardiomyopathy,^23^ obesity, hypertension and type 2 diabetes,^24^ type 2 diabetes, hypercholesterolaemia and osteopaenia^25^ and obesity, glucose intolerance and hyperinsulinaemia.^26^ Although individuals with SRS have been reported to be fertile^25 26^ with a low offspring risk of SRS,^19^ miscarriage, stillbirth and preterm neonatal death have been reported in women with SRS^26^ and genital anomalies in males and females are described which can impact on fertility.^19^

We have previously reported the ‘lived experience of SRS’ in a subset of individuals from the present cohort, emphasising that height was not the only major issue for adults with SRS and that there was a need for an adult service.^27^ To the authors’ knowledge, detailed health outcomes, quality of life and well-being have not been reported in a cohort of older individuals with SRS.

In this study of individuals aged 13 years and above with genetically confirmed SRS, we describe the adult phenotype and long-term outcome in terms of health and well-being, to develop a better understanding of the long-term prognosis of SRS.

## Methods

Informed consent was obtained from all participants. Participants were assessed in a research clinic by the same doctor (OL-S). History, clinical examination and growth parameters were recorded following a standardised in-depth interview framework, and childhood information was gathered from a parent using a standard questionnaire, either during the study appointment or by post. Hospital records were reviewed to confirm previous growth measurements, parental growth measurements, medical history and treatment.

### Study recruitment

Individuals with SRS aged ≥13 years, with matUPD7 or IC1 LOM were recruited: 1) via prior involvement in genetic research studies with the Wessex Imprinting Group, 2) following referral to diagnostic NHS Genetics Services or tertiary Paediatric Endocrine Centres within the UK, 3) through the UK Child Growth Foundation, 4) via the research study website. One participant was reported in a previous case report^25^ and it is likely that others participated in earlier UK childhood studies of SRS.^7 20^

### Phenotypic assessment using clinical scoring

We used three methods to score the adult cohort:

The NHCSS, based on six parameters measured at specific ages from birth to 3 years ((1) birth weight and/or length ≤−2 SDS; (2) height ≤−2 SDS at 2 years or height ≤−2 SDS from mid-parental target height; (3) relative macrocephaly; head circumference SDS ≥1.5 wt and/or length SDS at birth; (4) protruding forehead at 1–3 years; (5) body asymmetry; (6) feeding difficulties and/or low BMI (BMI ≤−2 SDS) at 2 years). Scores ≥4/6 suggest a clinical diagnosis of SRS. Scores of 3/6 are the threshold recommended for diagnostic genetic testing and defined as ‘possible SRS’.

Adult scoring method 1: we assessed five clinical parameters that are useful features in childhood scoring systems but based them on an examination as an adult: i) adult height ≤−2 SDS; ii) relative adult macrocephaly (head circumference SDS ≥1.5 height SDS); iii) protruding forehead as an adult; iv) body asymmetry in adulthood and v) feeding difficulties and/or low BMI (BMI ≤−2 SDS) in adulthood.

Adult scoring method 2: we assessed a mixture of findings at adult examination and past data from medical notes and parental questionnaires which were generally available for the majority of the cohort: i) birth weight and/or length ≤−2 SDS; ii) history of childhood feeding difficulties/low BMI; iii) adult height ≤−2 SDS, iv) relative adult macrocephaly (head circumference SDS ≥1.5 height SDS); v) protruding forehead in adulthood and vi) body asymmetry in adulthood.

Body asymmetry was defined as arm length or leg length discrepancy (LLD) of ≥0.5 cm or arm asymmetry or LLD <0.5 cm with at least two other asymmetrical body parts, with one being a non-face part.

### Growth

Birth weight, length and occipital-frontal circumferences were obtained from medical records or parent report. The interview included a validated puberty self-assessment questionnaire.^28^ At the research appointment, each participant’s height and weight were measured. Body mass index (BMI) was calculated as: weight (kg)/height (m^2^).

### Neurodevelopment and educational attainment

Developmental history was ascertained from parental reports and medical records. Educational attainment was reported by participants and/or their parents.

### Health, fertility and offspring risk

Information on medical problems was gathered from participants, their parents and medical notes.

Obesity was defined as a BMI of ≥30 kg/m^2^ or BMI SDS ≥2.^28^ A high waist circumference was defined as ≥94 cm in males (based on a Caucasian population); ≥80 cm in females (Caucasian and Asian populations).^29 30^ Blood samples were taken after at least 12 hours of fasting. A high triglyceride level was defined as ≥1.7 mmol/L or treatment for hypertriglyceridaemia.^30^ A high blood glucose was defined as fasting blood glucose ≥6.1 mmol/L; type 2 diabetes mellitus ≥7.0 mmol/L (fasting) or treatment for diabetes. Hypercholesterolaemia was defined as ≥5 mmol/L (as per generic National Health Service advice) or treatment for hypercholesterolaemia. Hypertension was defined as a systolic blood pressure ≥130 mm Hg and/or diastolic blood pressure ≥85 mm Hg (average of three examinations) or treatment for hypertension.^30^

### Quality of life and well-being assessment

A well-being questionnaire adapted for the study, the Sheehan Disability Scale tool^31^ and the ‘Schedule for the Evaluation of Individual Quality of Life-Direct Weighting’ (SEIQoL-DW) standardised assessment tool^32^ were administered face to face (see online supplementary information).

10.1136/jmedgenet-2019-106561.supp1 Supplementary data

### Molecular genetic analysis

Methylation at the imprinted differentially methylated regions (DMRs) of chromosome 7 and 11 (GRB10 alt-TSS DMR (7p12); MEST alt-TSS DMR (7q32), H19/IGF2 IG-DMR (or IC1, 11p15), KCNQ1OT1 TSS DMR (or IC2, 11p15)) was evaluated using methylation-specific PCR as previously described^33^ and methylation-specific multiplex ligation-dependent probe amplification^34^; findings of the two testing methods were fully concordant.

### Statistical analysis

SDS were calculated using the LMS growth Excel add-in and UK 1990 data^35^ for occipital-frontal circumference for age, height for age, weight for age and BMI for age. The upper age limits of the reference data for occipital-frontal circumference are 17 years and 18 years in females and males, respectively. The upper age limit for height and weight is 23 years. Participants’ SDS were calculated using their data for their actual age if within the reference data or, where the participant was older than the upper age limit, using the data for the highest age possible.

The distributions of continuous variables were examined for normality. Continuous variables with a normal distribution were compared between two groups using the two-sample t-test. Continuous variables with non-normal distributions were analysed using the Mann-Whitney U test, where there were two groups. Categorical variables were analysed using the Fisher’s exact or χ^2^ tests. Comparison of ordinal variables between two groups was performed using the Mann-Whitney U test.

## Results

Thirty-three patients were recruited including 18 (54.5%) females and 15 (45.5%) males with a median age of 29.58 years (range 13.36–69.71). IC1 LOM was identified in 27 (81.8%) and matUPD7 in 6 (18.2%). Results are provided by patient in the online supplementary table. These individuals make up the Study of Adults and Adolescents with Russell-Silver syndrome (STAARS) cohort.

### Growth

Amalgamated growth measurements of the cohort are shown by genotype (A) and by age (B) in table 1.

**Table 1:** Participant demographics and growth at the time of the study examination (unless indicated otherwise) of the STAARS UK cohort of 33 people with Silver-Russell syndrome

A) Data shown by genotype		
Phenotype	Genotype H19/IGF2 LOM	Genotype matUPD7
Number of patients (n, %)	27 (67.5)	6 (15.0)
**Gender**		
Male (n, %)	12 (44.4)	3 (50)
Female (n, %)	15 (55.6)	3 (50)
Age, years (median, IQR)	32.35 (13.32–69.71)	19.74 (14.47–33.93)
**Birth parameters (median, IQR), (n)**		
Gestation at birth, weeks	39 (37.0–40.6) (n=25)	38.0 (35.1–38.1)
Birth weight, g	1760 (1458–2098)	1805 (1505–2513)
Birth weight SDS	−3.54 (−4.20 to −2.64) (n=26)	−2.19 (−2.98 to −1.29)
Birth length, cm	40.6 (39.9–47.3) (n=10)	43.0 (n=1)
Birth length SDS	−4.06 (−5.26 to −0.55) (n=9)	−3.05 (n=1)
Birth head circumference, cm	33.8 (32.0–35.4) (n=8)	27.0 (n=1)
Birth head circumference SDS	−0.56 (−1.33 to 0.29) (n=8)	−0.79 (n=1)
**Growth parameters at examination (median, IQR, n=33)**		
Height, cm	153.0 (143.5–160.9)	156.8 (145.7–160.7)
Height SDS	−3.13 (−3.87 to −1.02)	−2.19 (−3.03 to −1.32)
Weight, kg	45.65 (38.90–62.30)	52.05 (45.38–56.81)
Weight SDS	−1.83 (−4.66 to −0.11)	−1.47 (−2.17 to −0.14)
BMI, kg/m^2^	19.7 (17.5–28.0)	22.9 (17.6–25.0)
BMI SDS	−0.80 (−1.99 to 1.49)	0.07 (−1.34 to 1.08)
**Growth hormone treatment**		
Yes (n, %)	17 (63.0)	6 (100)
No (n, %)	10 37.0)	0

B) Growth characteristics shown based on age; adolescents aged <18 years and adults ≥18 years				
	STAARS UK cohort	Individuals <18 years	Individuals ≥18 years	P value
n	33	8	25	
Gender				
Male	15 (45.5)	3 (37.5)	12 (48.0)	0.7
Female	18 (54.5)	5 (62.5)	13 (52.0)	
Age, years	29.58 (13.32–69.71)	14.15 (13.32–16.52)	32.88 (22.03–69.71)	
**Molecular genetic diagnosis**			
H19/IGF2 LOM	27 (71.8)	5 (62.5)	22 (88.0)	0.1
matUPD7	6 (18.2)	3 (37.5)	3 (12.0)	
**Growth parameters**				
Height SDS	−2.67 (−2.83 to −1.07)	−1.19 (−3.82 to −0.51)	−3.13 (−3.83 to −1.31)	0.1
Height in females, cm	146.2 (140.3 to 154.1) (n=18)	153.0 (130.9 to 154.2) (n=5)	144.7 (140.7 to 153.6) (n=13)	
Height in males, cm	159.4 (153.2 to 168.1) (n=15)	164.0 (median only as n=3)	158.1 (151.0 to 170.5) (n=12)	
Weight, kg	47.10 (41.65–58.38)	43.83 (31.68–48.26)	55.45 (43.85–63.42)	0.08
Weight SDS	−1.72 (−3.76 to −0.13)	−1.21 (−4.14 to −0.19)	−1.83 (−3.76 to −0.11)	0.95
BMI, kg/m^2^	20.5 (17.7–25.5)	17.0 (16.2–19.1)	21.2 (19.1–38.0)	0.003
BMI SDS	−0.53 (−1.83 to 1.13)	−1.33 (−1.84 to −0.58)	−0.47 (−1.83 to 1.53)	0.3
**Growth hormone treatment**				
Yes	23 (69.7)	8 (100)	15 (60.0)	0.07
No	10 (30.3)	0 (0)	10 (40.0)	

Growth parameters presented as median and IQR, n is the number of participants included where data are incomplete.

BMI, body mass index; LOM, loss of methylation; matUPD7, maternal uniparental disomy of chromosome 7; SDS, SD score; STAARS, Study of Adults and Adolescents with Russell Silver syndrome.

Historical data gathered during the interview showed that 78.8% (26/32) of individuals had a birth weight ≤−2 SDS. Relative macrocephaly at birth was present in 77.8% (7/9), where records were available. Preterm births occurred in 22.6% (7/31) of this cohort and the mean gestation of the cohort was 38 weeks (IQR 37–40). Intrauterine growth restriction was recorded in 76.7% (23/30) of pregnancies. The median age of puberty onset was 10.1 years in females and 12.3 years in males.

Measurements at the time of the study assessment showed that the median height SDS of the whole cohort was −2.67 (IQR −3.83 to −1.07); 60.6% (20/33) had a height SDS ≤−2 SDS despite GH treatment in 23 (69.7%) of the cohort. Males with IC1 LOM ≥18 years had a median final height of 156.9 cm (IQR 150.3–171.3) with a median height SDS of −3.13 (IQR −4.09 to −1.02). Females ≥18 years had a median final height of 144.7 cm (IQR 141.0–157.1) and a median height SDS of −3.17 (IQR −3.79 to −1.12). The final heights of individuals aged ≥18 years with matUPD7 was 159.3 cm (SDS −2.69) in the single male and a median of 143.6 cm (SDS −3.35) in the females (n=2). The median weight SDS was −1.72 and median BMI SDS −0.53. Median weight SDS and BMI SDS were −1.22 and −1.33, respectively, in males and −1.20 and −1.33 in females. The median head circumference was −0.95 SDS in adulthood and relative macrocephaly was present in 57.6%.

Asymmetry was present in 66.7% (22/33) and was observed more commonly in IC1 LOM cases than in matUPD7; 77.8% vs 16.7% (p=0.01).

### Adult dysmorphology

**Figure 1:**
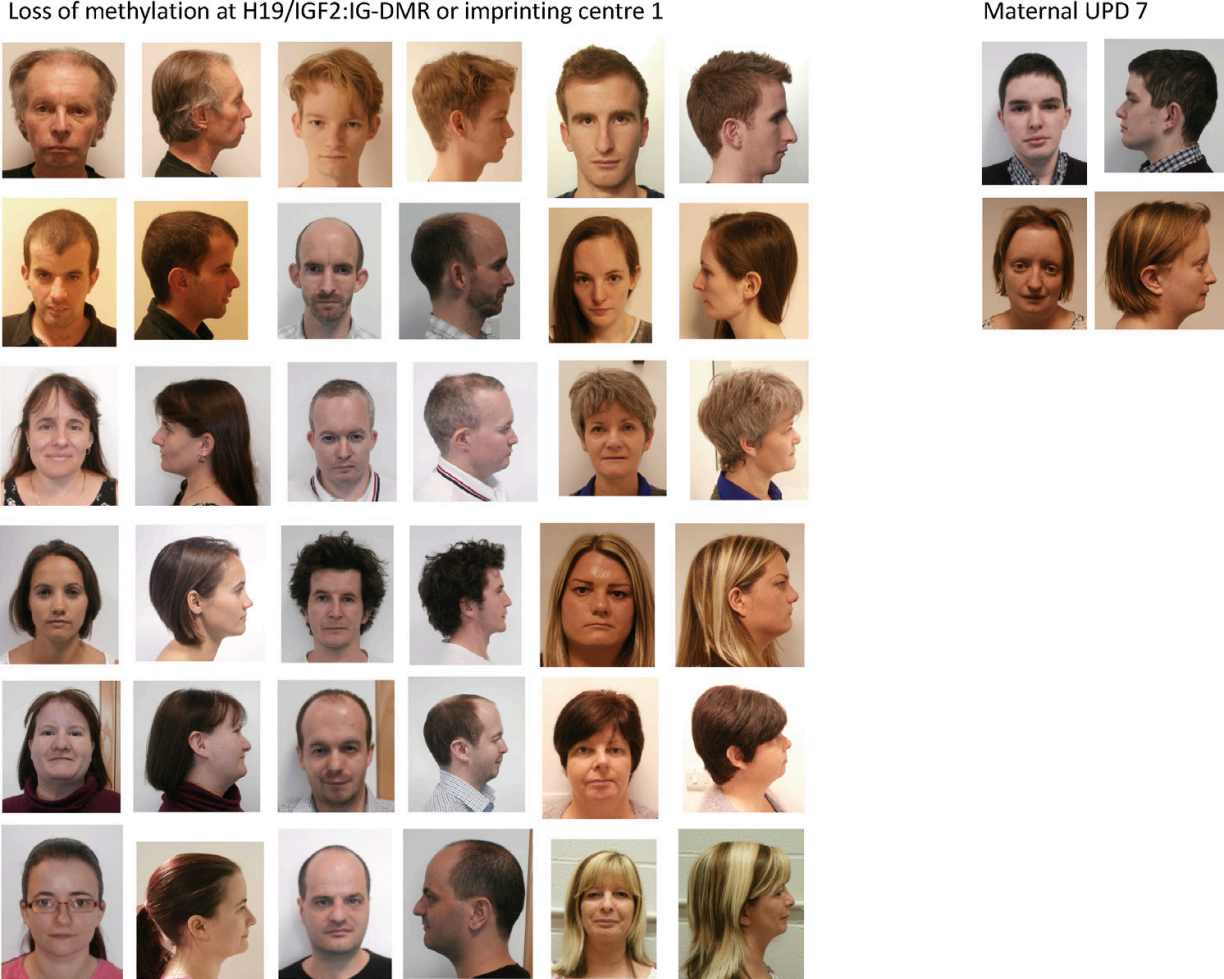
Adult phenotype of Silver-Russell syndrome by genotype. A broad forehead remains a facial feature in adults with H19/IGF2 loss of methylation and matUPD7 as shown in the photographs.

The adult facial appearance is shown in figure 1 and includes patients over the age of 18 years with consent to publish. A broad forehead and facial asymmetry (15/33) were useful diagnostic features when present. A triangular-shaped face, characteristic in childhood, was identified in only 25.8% (8/31), a broad nasal tip and broad nasal bridge were present in 21.2% (7/33) and 18.2% (6/33), respectively and retrognathia/micrognathia in 31.8% (7/22). Low-set ears and posteriorly rotated ears were present in 57.6% (19/33) and 54.5% (28/33), respectively. Down-slanting palpebral fissures were present in 30.3% (10/33).

### Relationship with food

While reported childhood feeding difficulties were a prominent feature (‘poor appetite’ (27/32), nasogastric tube feeding (19/32) and gastrostomy feeding (3/33)), reports of feeding issues in adulthood were largely unremarkable. They included two cases who described themselves as ‘constantly hungry’, seven cases in which the appetite was described as ‘good’ or ‘large’ or there was an allusion to eating large or excessive volumes, but four cases reported ‘fussy’ or ‘difficulty with’ eating.

### Neurodevelopment and educational attainment

Concerns about early development were reported with delay reaching motor milestones in 64.5% (20/31) and speech development in 38.7% (12/31). The median age for walking was 16 months (IQR 13–24, n=27). Speech therapy had been received in 18.2% (6/33). The majority of individuals had attended mainstream education, but special educational support had been accessed in nine cases at some point during their education; mainstream primary school with special educational support in 21.2% (7/33), mainstream secondary with special educational support in 21.2% (7/33) and secondary school for children with special educational needs in 6.1% (2/33).

General Certificates of Secondary Education (GCSEs) or equivalents, including Certificates of Secondary Education and General Certificates of Education Ordinary level (O-levels), were attained in 92.6% (25/27) of cases aged ≥16 years. General Certificates of Education Advanced levels (A-levels) or equivalents, including Business and Technology Education Council (BTEC) qualifications, were gained in 56.0% (14/25) of cases aged ≥18 years. University degrees were completed in 40.0% (10/25) of cases aged ≥21 years and one degree-level BTEC was achieved. There was no association between historical reported concern about attainment of normal motor milestones (as reported by parents) and GCSE attainment (p=0.326), although A-level attainment was less likely where there had been significant concerns (25.0% vs 72.7%, p=0.014).

### Phenotypic assessment using different clinical scoring systems

Insufficient historical data were available to score the majority of patients using the NHCSS (data not shown). The results of the novel two ‘adult’ scoring methods are shown in table 2 and are based on a cohort of 29 cases where all parameters were available. Twenty per cent of participants had a score of 4 or above/5 and ~50% had a score of 3 or above using method 1. When available historical data on birth weight and childhood feeding difficulties were included, ~40% had a score of 4 or above/6 and ~70% had a score of 3 or above.

**Table 2:** Results of the clinical assessment of particular phenotypes in adulthood to create two novel scoring systems method

Molecularly confirmed patients with SRS assessed using two new adult scoring systems	Percentage of patients scoring (≥4)	Percentage of patients scoring (≥3)
Method 1 based on five criteria (n=29)	21% 6/29	48% 14/29
Method 2 based on six criteria (n=29)	41% 12/29	69% 20/29

Method 1 assessed i) adult height ≤ −2 SDS, ii) relative adult macrocephaly (head circumference SDS ≥1.5 length SDS, iii) protruding forehead in adulthood, iv) body asymmetry in adulthood and v) feeding difficulties and/or low BMI (BMI ≤ −2 SDS) in adulthood.

Method 2 used historical data from medical notes and parental questionnaires available in the majority of adults in addition to examination and assessed i) birth weight and/or length ≤ −2 SDS, ii) history of childhood feeding difficulties/low BMI, iii) adult height ≤ −2 SDS, iv) relative adult macrocephaly (head circumference SDS ≥1.5 length SDS), v) protruding forehead in adulthood, vi) body asymmetry in adulthood.

BMI, body mass index; SDS, SD score; SRS, Silver-Russell syndrome.

### Health problems

#### Congenital anomalies

Congenital anomalies were present in 54.5% (18/33) of cases (table 3) and included abnormal development of the palate, scoliosis, genital anomalies, congenital heart disease and renal anomalies in more than one patient. Of the 18 cases in which congenital anomalies were found, 50.0% (9/18) had a single anomaly, 22.2%^4^ had two, 16.7%^3^ had three, four in one (5.6%) and five in one (5.6%). Apart from two congenital anomalies in one individual with matUPD7, all congenital anomalies were found in individuals with IC1 LOM.

**Table 3:** Congenital anomalies identified in the cohort of 33 patients by genotype

Phenotype	All cases (n=33)	H19/IGF2 LOM (n=27)	matUPD7 (n=6)	P value
Congenital anomaly present	54.5%	63.0%	16.7%	0.07
Cleft palate/Bifid uvula	9.1%	11.5%	0%	0.6
Female genital anomalies	16.7% (n=18)	20.0% (n=15)	0% (n=3)	0.6
Male genital anomalies	33.3% (n=15)	41.7% (n=12)	0% (n=3)	0.5
Cardiac anomalies	3	2	1	
Brain anomalies	1	1	0	
Renal anomalies	3	2	1	
Radial anomalies	1	1	0	
Thumb anomalies	2	2	0	
Coloboma	1	1	0	
Scoliosis/Kyphoscoliosis	8	7	1	0.7
Limited elbow supination/Congenital dislocation	3	3	0	0.6
Camptodactyly	5	5	0	0.6

Radial and thumb anomalies included hypoplasia of the radii with absent thumbs bilaterally, a bifid thumb, congenital dislocation of the radial head. Of the three individuals with cleft palate, a bifid uvula only was present in one of these cases. The genital anomalies in females included: 1) vaginal agenesis with a hypoplastic uterus and single ovary; 2) hypoplastic genitalia with pronounced labia minora and a history of vaginal hernia and 3) a bicornuate uterus with double cervix. The genital anomalies in males included: 1) a history of bilateral cryptorchidism in four cases and 2) a history of ambiguous genitalia and severe hypospadias. The cardiac anomalies were 1) tricuspid valve regurgitation; 2) cardiac juxtaposition and 3) history of coarctation of the aorta with multiple ventriculo-septal defects. The renal anomalies reported were 1) a solitary kidney with crossed fused ectopia; 2) horseshoe kidney and 3) malrotation of one kidney. There was one case of ‘congenital hip dislocation’. The coloboma was of the iris only. The brain abnormality was reported as a dysplastic corpus callosum.

LOM, loss of methylation; matUPD7, maternal uniparental disomy of chromosome 7.

#### Musculoskeletal problems

Myalgia and arthralgia of the back, hip, neck, knees and fingers affected 27.3% (9/33) of participants, eight of whom were women. The severity of pain affected mobility in two individuals, who each used a wheelchair for travelling long distances; 44.4% (8/18) of females reported joint pains and aches compared with 6.66% (1/15) of males (p=0.021). Further musculoskeletal problems included hypermobility (n=2), trigger finger (n=1), Raynaud’s syndrome (n=2), a ganglion cyst (n=1), childhood rheumatoid arthritis (n=1), anterior cruciate ligament tear (n=1), locking knees (n=1), joint dislocations (n=3), pes cavus (n=2), osteopaenia (n=2), osteoarthritis (n=1), patella alto (n=1) and prolapsed vertebral disc (n=1). In six cases, there were more than one co-existing.

#### Respiratory problems

Lung disease was reported in 36.4% (12/33): asthma (n=9), restrictive lung disease (n=1), bronchiectasis (n=1), primary pulmonary hypertension (n=1), chest infections (n=1).

#### Allergy

There was a history of food allergy or intolerance in 15.2% (5/33). Two of these individuals had peanut allergy; one an idiopathic food allergy; two had dairy and soya intolerance with one of these individuals also having an intolerance to egg. Three individuals reported a history of hay fever. A history of skin conditions was noted in 18.2% (6/33) with eczema in three individuals, acne in two individuals, psoriasis in one and a history of both eczema and acne in another case.

#### Gastrointestinal disorders

These were diagnosed in 18.2% (6/33). Gastro-oesophageal reflux remained a problem in five individuals, one of whom had been demonstrated to have delayed gastric emptying. Irritable bowel syndrome was diagnosed in one case and was a possible diagnosis in another.

#### Cardiometabolic health/outcomes

A history of hypoglycaemia was reported by the participant or parent in 58.6%. In the cohort overall, obesity was present in 9.1%. The median waist-to-hip ratios in females and males were 0.820 and 0.890, respectively. Median triglyceride concentration was 1.00 mmol/L with a raised triglyceride level in 16.1%. Median fasting glucose level was 4.8 mmol/L. Ten per cent of subjects had type 2 diabetes. Hypertension was present in 27.6% and hypercholesterolaemia in 43.8% of subjects (table 4).

**Table 4:** Cardiometabolic health parameters in cohort as a whole and for the subgroup aged ≥18 years only

	All	Aged ≥18 years only
N	33	25
Female, n (%)	18 (54.5)	13 (52.0)
Male, n (%)	15 (45.5)	12 (48.0)
BMI SDS (median, IQR)	−0.53 (−1.83 to 1.14)	−0.47 (−1.83 to 1.53)
Obesity	9.1% (3/33)	12.0% (3/25)
High waist circumference	30.3% (10/33)	36.0% (9/25)
Waist-to-hip ratio in females (median, IQR)	0.820 (0.762–0.893) (n=18)	0.826 (0.767–0.893) (n=13)
Waist-to-hip ratio in males (median, IQR)	0.890 (0.834–0.973) (n=15)	0.932 (0.883–0.977) (n=12)
DXA total fat percentage (median, IQR)	41.31 (29.53–46.88) (n=22)	44.45 (31.45–46.88) (n=18)
DXA subtotal fat percentage (median, IQR)	42.77 (29.83–48.42) (n=22)	46.09 (32.03–48.42) (n=18)
Triglyceride level, mmol/L (median, IQR)	1.00 (0.80–1.50) (n=31)	1.05 (0.80–1.58) (n=24)
High triglyceride level (≥1.7 mmol/L)	16.1% (5/31)	20.8% (5/24)
Glucose level, mmol/L (median, IQR)	4.8 (4.4–5.7) (n=30)	4.95 (4.40–6.18) (n=24)
High blood glucose (≥6.1 mmol/L)	20.0% (6/30)	25.0% (6/24)
Type 2 diabetes mellitus	10.0% (3/30)	12.5% (3/24)
Total cholesterol level, mmol/L (median, IQR)	4.80 (4.30–5.63) (n=32)	5.00 (4.30–5.75) (n=25)
Hypercholesterolaemia (≥5 mmol/L)	43.8% (14/32)	52.0% (13/25)
Hypertension (Alberti criteria 130/85)	27.6% (8/29)	33.3% (8/24)

DXA, dual energy X-ray absorptiometry.

In the cohort restricted to subjects ≥18 years, six participants had evidence of impaired glucose tolerance (25% (6/24)); type 2 diabetes was reported in three people aged 69, 56 and 37 years (latter diagnosed as a result of this study), one patient had impaired glucose tolerance (female 36.3 years) and two had impaired fasting glycaemia (male 33 years; female 33 years). Other evidence of metabolic disturbance in those aged ≥18 years included a raised cholesterol in 52.0% (13/25) and there was hypertension in 8 of 24 (33.3%).

#### Unexplained drop/dizzy attacks

There was a history of unexplained dizziness, faints and recurrent collapses in four individuals (12.1%), one of whom was reported to have postural hypotension. There was no common precipitating time or reason for these observations. One individual had been extensively investigated and hypoglycaemia excluded.

#### Issues warranting surgery

In addition to surgery to correct the congenital anomalies presented in table 4, there were additional surgical interventions in 36.4% (12/33). These included surgery for herniae (n=3) (two specified as inguinal), leg lengthening (n=2) and subsequent femoral epiphysiodesis in one of these cases, ligament and/or tendon surgery (n=2), pinnaplasty (n=3), gastrostomy and reversal (n=2), rhinoplasty (n=1), sterilisation (n=1) and jaw surgery (n=1). One individual had a history of malignant hyperthermia at induction of anaesthesia.

#### Other health problems

Dental intervention was reported in 48.5% (16/33) with braces having been required in 7 cases, tooth extraction in 12 cases, dental operations reported in 1 case and unspecified treatment in 1 case. Five participants gave a history of migraine (n=3) or headaches (n=2).

### Fertility and offspring risk

Nine (five females and four males) out of 25 participants aged ≥18 years had children (three had one child, four had two children and two had three children). Eight of these individuals had IC1 LOM and one had matUPD7. None of the offspring were affected with SRS.

One male with a history of cryptorchidism was father to two children. One further male, who had been diagnosed with testosterone deficiency but with normal follicle-stimulating hormone and luteinising hormone, had one offspring. Of the other 11 males, primary hypogonadism, azoospermia and infertility had been confirmed in 1 male, who had a history of severe hypospadias at birth. One of the four males with a history of cryptorchidism and orchidopexy was noted to have small testicular volumes during puberty, a borderline testosterone level and a raised follicle-stimulating hormone. In addition to the three females with genital anomalies (3/18) presented in table 4, there was a history of gynaecological problems in 22.2% (4/18) of females, including endometriosis (n=1), menorrhagia (n=2) and pelvic inflammatory disease (n=1).

### Life satisfaction, disability and quality of life

Overall, the median score of the life satisfaction ladder was 8.0 out of 10.0 (IQR 7.0–8.0). Scores on the ladder range from 0 to 10, where 0 is the worst possible life a participant could imagine and 10 is the best. There was no difference between GH-untreated (n=10) and GH-treated (n=23) individuals in their life satisfaction ladder scores; 7.5 (IQR 4.0–8.4) and 8.0 (IQR 7.0–8.0), respectively (p=0.340), although the range of answers was greater in the untreated group. There were no differences in the descriptions of health (p=0.655), feelings about school/job (p=0.573), feelings of being an outsider or left out (p=0.899), feeling awkward and out of place (p=0.488), or feeling lonely (p=0.771) between GH treatment groups.

In the cohort overall, the median total Sheehan Disability Scale score was 3 out of 30% and 33.3% gave a score of 0 (ie, no disability).

The quality of life questionnaire produced a mean SEIQoL-DW index score of 74.9, which is comparable to that of 77.4 (SD 9.5) obtained in healthy adults.^31^ It revealed no significant correlation between SEIQoL-DW index score and height SDS (Pearson’s correlation coefficient 0.117, p=0.529). However, there was a negative correlation between SEIQoL-DW index score and BMI SDS (Pearson’s correlation coefficient −0.388, p=0.031).

## Discussion

To our knowledge, this is the largest cohort studied of older people with molecularly confirmed SRS. Our study addresses the paucity of information for families and health professionals on the adult phenotype and long-term prognosis of SRS. Only cases with a confirmed molecular diagnosis were included, to minimise heterogeneity. The use of retrospective reports for early growth and development measurements meant that we were able to confirm that the cohort was comparable to previously reported childhood cohorts,^7 18 20^ with a typical prevalence of IC1 LOM and matUPD7. Our results are therefore a useful representation of the long-term outcome of SRS, even though the considerable age range of patients means that some received treatments that differ from those recommended today.

Diagnosis of SRS in adulthood is difficult and our results show that many of the diagnostic characteristics typical in childhood, such as feeding difficulties, triangular facial appearance and relative macrocephaly were no longer useful diagnostic discriminators of SRS in adulthood. The NHCSS for SRS, an internationally agreed diagnostic score for use in childhood^19^ could not be used on our cohort as insufficient data were available despite referring back to medical notes. We found that head circumference in particular was not routinely measured in the UK and appropriate photos were not always available. It shows the importance of recording birth head circumference (and ideally birth length), especially for small newborns. It is done in many countries and should be performed in every country when babies are born in hospital. We attempted to adapt the criteria for adulthood but we were not able to create a score that was sensitive enough to be useful despite all cases having a proven molecular aetiology for SRS. Our study addressed the uncertainty as to the applicability of NHCSS in older people. It provides evidence that the NHCSS has to be strictly applied at the ages specified during childhood and that use of this scoring system using adult data is not appropriate. Two of the six diagnostic parameters in childhood involve head growth (relative macrocephaly and a prominent forehead in childhood). In this adult cohort, relative macrocephaly at examination based on strict criteria was only seen in 58%, compared with 86% usually seen in childhood.^19^ This means that this measurement does not replicate the finding in childhood. This is likely to be due to in utero growth restriction sparing head growth, with reduced differential growth postnatally. Protuberance of the forehead is likewise no longer a significant finding in adulthood and can only be assessed retrospectively if appropriate photos are available. Another criterion, feeding difficulty, is not a significant issue in adults. In this cohort, despite an early history of poor appetite in 84.4%, feeding problems at the time of the research appointment related mainly to excessive intake. These findings mirror the observation that feeding problems and the requirement for enteral feeding in SRS reduce with increasing age.^5^ However, in this cohort, proportionate short stature and body asymmetry remain key features useful in diagnosis, particularly in IC1 LOM cases where somatic mosaicism is likely to be the cause of asymmetric growth of body parts. Both of these parameters, however, are amenable to treatment and it is important for health professionals to appreciate that a specific clinical diagnosis is much more difficult for patients presenting in adulthood and genetic testing should be considered at an earlier stage in the diagnostic pathway for people presenting with possible SRS in later life. Arguably, during adulthood, SRS should therefore be a molecular diagnosis and this issue should be discussed in the next revision of the international SRS consensus.

This cohort identifies short stature as a major finding in adults with SRS with only 40% above −2 SDS for height. In the subgroup aged ≥18 years, despite ~70% having received GH, final height remained restricted (−3.13 SDS). It is important to note that the exact indication and regimens could not be elucidated and that current practice is different to historical cases. A combination of GH and gonadotrophin-releasing hormone analogues have provided improved height gain in a Dutch cohort of 17 patients^6^ and are recommended in the consensus guidelines of 2017.^19^

Our cohort highlights that older people with SRS often have diverse medical issues requiring ongoing access to medical services into adulthood despite the absence of a control population to compare representation of illnesses. Further study is warranted to determine if the findings are generalisable. Abnormal glycaemic control was a feature in 25% of people aged ≥18 years and the high levels of hypercholesterolaemia and hypertension provide some evidence of metabolic dysfunction in adults with SRS, even without obesity, and warrants further investigation. Additional health surveillance for these outcomes may be required for older people with SRS to allow for preventive measures. Arthralgia in female patients with SRS was an unexpected finding, and may have been exacerbated by long-standing low muscle tone and/or joint hyperextensibility. This finding echoes our previous lived experience work, in which women reported pain and disability and the impact this had on relationships and employment.^27^

Drop attacks/dizzy episodes were reported in four people which has also been reported by individuals in the USA (MAGIC foundation, personal communication). One patient had been extensively investigated without determining an underlying cause. It is unclear whether these attacks have related causes to the unexplained excessive perspiration^36 37^ or diaphoresis and ‘pale’ episodes during early life reported by 52% of parents of children with a clinical diagnosis of SRS.^20^ These historical events were largely not investigated. One hypothesis is that they relate to hypersensitive vagal episodes induced by gut dysmotility.

This cohort included four men and five women with SRS who have had children, providing reassurance about normal fertility for patients growing up with this condition, although it is important to acknowledge that some patients with SRS have genital anomalies/reproductive anatomy incompatible with fertility. The fact that no offspring have SRS is in keeping with the literature of low recurrence risk for SRS in the genetic subtypes represented in this study. However, the risk will depend on the underlying genetic cause.^19^ Molecular testing is important for all patients with a potential diagnosis of SRS who are planning to start a family.^7^

Educational attainment has not previously been reported in an adult SRS cohort. Forty per cent of the cohort attended tertiary education in line with general UK attendance of 42% in non-SRS populations^38^ providing evidence of a normal expectation of education attainment for the majority. This is supported by results from a new study of 10 patients with SRS who have been shown to have average intelligence (assessed using the full-scale IQ).^39^ While there were early reports of delay with development, only two participants attended a secondary school for those with special educational needs.

The Cantril ladder of life measure has been used in international surveys and results summarised in the World Happiness report.^40^ The average UK score of 6.714 is below that seen in the UK STAARS cohort. Low levels of disability were also reported in this cohort. Interestingly, there was no improvement in quality of life with increasing height but it was shown to reduce with increasing BMI. This highlights the importance of issues other than height in the management of SRS adding support to our previous observations that people living with SRS experience challenges that extend beyond a concern about height.^27^

There are important limitations of our study that should be discussed. The retrospective nature of the study means that: 1) participant and parental recall may not have been accurate; 2) current growth management of SRS is likely to differ from historical treatments received by older participants. Furthermore, individuals with SRS who took part in this study may not precisely represent a group with the health of all individuals with SRS or different genetic subgroups. It is possible therefore, that our subjects differ from others with SRS (eg, a subject’s medical problems may alter their willingness to participate in research). Furthermore, the life satisfaction, disability and quality of life measures show scores equal to an unselected population, yet our previous qualitative data provided more contextual and descriptive information regarding the psychosocial challenges experienced by participants during childhood, adolescence and beyond. One reason for this mismatch might be that quantitative measures capture a ‘snapshot’ of how a participant feels ‘at the present time’ or in the last week, whereas in-depth interviews cover a much longer time frame and in more detail.

## Conclusion

Our results show that many of the diagnostic characteristics typical in childhood, such as feeding difficulties, triangular facial appearance and relative macrocephaly were no longer useful diagnostic discriminators of SRS in adulthood. Our study shows that past treatment regimens have not prevented significant short stature in adults with SRS and has identified medical issues warranting further research, particularly predisposition to diabetes. However, it is clear from this study that SRS is compatible with long-term well-being, normal school attainments, family life and a quality of life equal to individuals with normal stature.
