# Supplementary material for: Phenotype of genetically confirmed Silver-Russell syndrome beyond childhood
Source: J Med Genet. 2020 Feb 13;57(10):683–91. doi: 10.1136/jmedgenet-2019-106561 (PMC7525777; doi:10.1136/jmedgenet-2019-106561)
Supplement: Supplementary data [file jmedgenet-2019-106561supp001.pdf]

	Sex	Age	Epigenotype	Birth weight (g)	Birth length (cm)	Birth head circumference (cm)	Gestation (weeks)	Nasogastric tube feeding	Gastrostomy	History of a poor appetite	Growth hormone treatment	Number of offspring	low BMI as defined by NHCSS (<= -2 SDS)	Head circumference (cm)	Height (cm)	Weight (kg)	Waist circumference (cm)	Sitting height (cm)	Hip circumference (cm)	Asymmetry as defined by NHCSS	GCSEs or equivalent	A-levels or equivalent	Degrees or equivalent
1	male	32.62	hypomethylation ICR1/H19	2070	48.26	.	38.29	yes	no	yes	Yes	2	no	56	171.9	83.7	101.33	94.17	104.13	yes	yes	yes	yes
2	female	37.19	hypomethylation ICR1/H19	2665	.	.	.	no	no	yes	No	2	no	56.4	149.53	65.15	88.17	79.5	108.27	yes	yes	no	no
3	female	16.89	matUPD7	1710	.	.	36.86	yes	yes	yes	Yes	0	no	57.7	154.37	58.95	91.8	80.5	102.57	yes	yes	no	no
4	female	29.58	hypomethylation ICR1/H19	2270	.	.	40.57	no	no	yes	Yes	0	no	54.2	160.87	54.65	70.33	85.37	93.63	no	yes	yes	yes

5	female	36.3	hypomethylation ICR1/H19	800	.	.	42	yes	no	yes	No	0	no	52.4	130.57	64.53	89.4	74.9	114.13	no	yes	no	no
6	male	32.88	hypomethylation ICR1/H19	1760	43.5	35.5	40	yes	no	yes	Yes	0	no	56.5	153.23	65.85	97.13	77.67	101.9	yes	yes	no	no
7	male	34.03	hypomethylation ICR1/H19	2240	50	.	37	yes	no	yes	No	1	no	55.6	171.25	62.3	79.23	90.15	89.77	yes	yes	yes	yes
8	male	40.25	hypomethylation ICR1/H19	1310	38	32	38	yes	no	yes	Yes	0	yes	54.1	146.07	37.1	66.27	80	74.83	yes	yes	no	yes
9	male	37.09	hypomethylation ICR1/H19	1250	.	.	37	yes	no	yes	Yes	0	no	55.3	150.3	44.1	84.33	81.43	85.6	yes	yes	yes	yes
10	male	22.03	matUPD7	1644	.	.	38	.	no	yes	Yes	0	no	57.8	159.3	55.45	79.13	87.37	88.93	no	yes	yes	no
11	female	26.09	hypomethylation ICR1/H19	1740	40.5	.	37.14	yes	no	no	No	1	no	55.2	144.7	44.25	71.3	78.45	87.57	no	yes	yes	no

12	male	39.14	hypomethylation ICR1/H19	1871	40.64	.	40	no	no	no	No	3	no	57.2	156.85	75.45	99.6	89.4	107.45	yes	no	no	no
13	male	14.47	matUPD7	2435	.	.	38	no	no	yes	Yes	0	no	55.4	159.4	42	60.25	84.45	76.25	no	no	no	no
14	female	13.75	hypomethylation ICR1/H19	1065	.	28	33.57	yes	no	yes	Yes	0	no	53.4	153	45.65	65.65	84.75	88.45	yes	no	no	no
15	female	25.11	matUPD7	1899	43	.	38	no	no	yes	Yes	0	no	55.9	147.7	56.1	76.1	83.35	103.85	no	yes	no	no
16	male	23.44	hypomethylation ICR1/H19	1645	.	32	36	yes	yes	.	Yes	0	yes	56.1	163	41.3	66.45	84.95	76.15	yes	yes	yes	no
17	female	47.86	hypomethylation ICR1/H19	2098	.	.	42	no	no	yes	No	2	no	51	143.45	57.7	94.47	78.65	98.37	yes	yes	no	no
18	female	33.93	matUPD7	1088	.	27	30	no	no	yes	Yes	2	no	54.2	139.5	46.5	80.55	77.7	90.3	no	yes	yes	no
19	female	28.64	hypomethylation ICR1/H19	946	.	.	33	no	no	yes	No	0	yes	52.1	144.05	35	60.15	80.7	82.35	no	yes	yes	no

20	male	69.71	hypomethylation ICR1/H19	1871	.	.	42	no	no	yes	No	1	yes	53.8	153.9	43.6	78.1	81.6	79.8	yes	no	no	no
21	male	17.44	matUPD7	2745	.	.	38.57	yes	no	no	Yes	0	no	55.1	164.55	48.65	69.4	87.95	83.35	no	yes	no	no
22	female	33.01	hypomethylation ICR1/H19	2200	47	.	37	no	no	yes	No	0	no	54.7	157.1	83.75	105.8	84.85	118.25	no	yes	no	no
23	female	56.85	hypomethylation ICR1/H19	1760	.	.	41.43	no	no	yes	Yes	3	no	53	140.95	44.35	79.85	78.25	88.3	yes	yes	no	yes
24	male	27.29	hypomethylation ICR1/H19	2350	.	34.5	40	no	no	yes	Yes	0	yes	56.1	171.95	55.5	79.1	90.55	84.33	yes	yes	yes	yes
25	female	13.83	hypomethylation ICR1/H19	1870	.	.	39.86	yes	yes	yes	Yes	0	yes	51.7	126.9	22.7	52.23	.	65.6	yes	no	no	no
26	male	32.35	hypomethylation ICR1/H19	2041	.	.	38	yes	no	yes	Yes	0	no	57.7	168.05	57.8	76.1	.	91.3	yes	yes	yes	yes

27	female	26.99	hypomethylation ICR1/H19	1730	.	.	40.57	yes	no	yes	Yes	0	no	52.7	140.5	38.9	70.25	.	85.05	yes	yes	yes	yes
28	male	31.28	hypomethylation ICR1/H19	1560	40.6	35	39	yes	no	yes	Yes	0	no	56.3	136.47	36.65	76.35	75.2	75.35	no	yes	no	no
29	female	24.4	hypomethylation ICR1/H19	1304	38	.	.	yes	no	no	No	0	no	55.2	157.07	44.4	74.75	.	90.35	yes	yes	yes	yes
30	female	13.32	hypomethylation ICR1/H19	1460	.	.	35	yes	no	yes	Yes	0	no	55.5	153.95	38.5	64	.	83.6	yes	no	no	no
31	female	54.7	hypomethylation ICR1/H19	1760	40.6	35.6	40.43	yes	no	yes	Yes	0	no	53.9	150.1	57.1	84.45	.	99	yes	yes	yes	no
32	female	15.4	hypomethylation ICR1/H19	1458	.	33	38.71	yes	no	yes	Yes	0	no	52.7	134.9	29.4	65.4	.	76.55	yes	no	no	no
33	male	13.83	hypomethylation ICR1/H19	2650	.	.	42	no	no	no	Yes	0	no	52.8	164	47.1	64.25	.	83.6	yes	no	no	no
